# The Use of Sub-Mental Ultrasonography for Identifying Patients with Severe Obstructive Sleep Apnea

**DOI:** 10.1371/journal.pone.0062848

**Published:** 2013-05-10

**Authors:** Chin-Chung Shu, Peilin Lee, Jou-Wei Lin, Chun-Ta Huang, Yeun-Chung Chang, Chong-Jen Yu, Hao-Chien Wang

**Affiliations:** 1 Department of Traumatology, National Taiwan University Hospital, Taipei City, Taiwan; 2 School of Medicine, College of Medicine, National Taiwan University, Taipei City, Taiwan; 3 Department of Internal Medicine, National Taiwan University Hospital, Taipei City, Taiwan; 4 Center of Sleep Disorder, National Taiwan University Hospital, Taipei, Taiwan; 5 Department of Internal Medicine, National Taiwan University Hospital, Yun-Lin Branch, Yun-Lin County, Taiwan; 6 Department of Medical Imaging, National Taiwan University Hospital, Taipei, Taiwan; The Chinese University of Hong Kong, Hong Kong

## Abstract

**Objective:**

This study aimed to explore the association between obstructive sleep apnea (OSA) severity and pharyngeal parameters using sub-mental ultrasonography (US), and investigate the accuracy of US for identifying severe OSA patients.

**Methods:**

One hundred and five consecutive referrals for suspected OSA were enrolled. The diameters of the retro-glossal (RG) and retro-palatal (RP) regions were measured via sub-mental US upon expiration during tidal breathing, forced inspiration, and Müller maneuver (MM). Independent factors associated with severe OSA identified from two-thirds of randomly-selected patients (model-development group) were used to construct a model for predicting severe OSA. The accuracy of the model was validated in the remaining one-third of patients (validation group).

**Results:**

Fifty severe OSA patients, 30 with mild-moderate OSA, and 25 without OSA were enrolled. Compared to non-OSA and mild-moderate OSA patients, those with severe OSA had narrower RP diameter in all three maneuvers. Using the prediction model constructed with changes of RP diameters at MM and neck circumference, the independent predictors of severe OSA in the model-development group had 100% sensitivity and 65% specificity.

**Conclusion:**

Sub-mental US can accurately discriminate the severity of OSA and be used to identify patients with severe OSA.

**Trial Registration:**

ClinicalTrials.gov NCT00674076

## Introduction

Obstructive sleep apnea (OSA) is defined as the collapse of the upper airway during sleep, causing intermittent hypoxia and sleep fragmentation [1]. It can result in cardiovascular diseases, metabolic dysregulation, and neuro-cognitive dysfunction [2]–[5]. Early diagnosis and intervention with continuous positive airway pressure (CPAP) can effectively improve airway patency, daytime wakefulness, blood pressure, metabolic abnormalities, and quality of life [6]–[8].

Currently, the diagnosis of OSA is based on clinical symptoms and episodes of apnea and hypopnea measured by polysomnography (PSG) [9]. However, PSG has limited availability. A questionnaire on sleep disorder and overnight pulse oximetry are used for screening before PSG [10], [11]. The questionnaire has 77% sensitivity and 53% specificity for predicting OSA [10]. In severe OSA patients [8], the combination of questionnaire and pulse oximetry has 85% sensitivity [12]. For better prediction of severe OSA, radiographic modalities such as magnetic resonance imaging (MRI), computed tomography (CT), and ultrasonography (US) have been applied to assess the upper airway anatomy in OSA patients [13]–[15]. However, MRI and CT cannot be widely applied due to the high costs and radiation exposure, respectively. In contrast, US is radiation-free, less expensive, and portable, thereby allowing for high accessibility for screening purposes.

A previous work has demonstrated that US can clearly demonstrate the vocal cords and air column of the upper airway where it can be used to identify patients at high risk for post-extubation stridor [16]. Several studies also demonstrate the role of US in detecting anatomic risk factors for OSA, including para-pharyngeal pad and tongue [17]–[19]. However, these studies have mainly focused on anatomic characteristics without addressing the dynamic changes of airspace that may better reflect the influence of neurologic control. We hypothesized that detecting both the anatomic characteristics and the dynamic changes of pharyngeal airspace by sub-mental US can discriminate OSA severity and be used for identifying severe OSA patients. The present study therefore aimed to identify patients with severe OSA using the dynamic changes of pharyngeal airspace on expiration during tidal breathing, forced inspiration, and the Müller maneuver.

## Materials and Methods

### Participants

This study was performed at the Center of Sleep Disorder of National Taiwan University Hospital from January to June 2008. All Chinese patients aged ≥18 years-old and referred to the sleep laboratory for suspected OSA were eligible for the study. The exclusion criteria were refusal to participate; inability to perform the maneuvers; presence of congestive heart failure, chronic pulmonary disease demonstrated on pulmonary function testing, active neurologic event, active infection or surgery two weeks prior to screening; enrolment in other trials during the study period; other sleep disorders; ascites; and pregnancy. Clinical evaluation, anthropometric measurements, and US examination were immediately conducted upon enrolment whereas PSG was undertaken within three days after US.

Two-thirds of the participants were randomly selected as the model-development group while the remaining one-third was the validation group. The allocation was based on a computer-generated random assignment. The Research Ethics Committee of National Taiwan University Hospital approved the study protocol and all participants provided written informed consent.

### Polysomnography

Full-night PSG (Embla N7000, Medcare Flaga, Reykjavik, Iceland) was performed in the sleep laboratory according to a previous protocol [20]. Apnea was defined as the absence of airflow ≥10 sec and hypopnea was >50% decrease in airflow ≥10 sec associated with reduced arterial oxygen saturation in 4%, or an arousal. An independent technician blinded to the US results analyzed all of the sleep studies. Non-OSA was defined as apnea-hypopnea index (AHI) <5/hr, mild-moderate OSA as AHI between 5–30/hr, and severe OSA as AHI ≥30/hr.

### Sub-mental US

**Figure 1:**
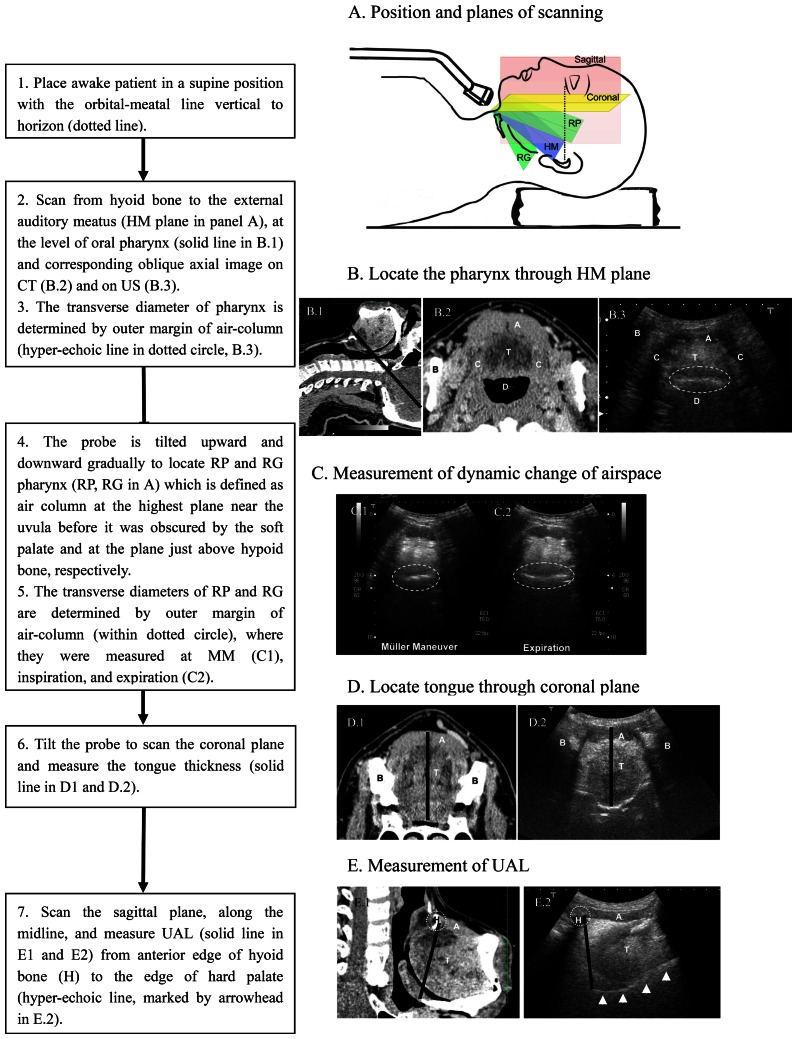
Protocol for sub-mental scanning. The procedure on ultrasound scanning is shown on the left panel and the corresponding computed tomography and ultrasonographic images on the right panel. A, Geniohyoid muscle; B, ramus of the mandible; C, hypoglossal muscle and corresponding acoustic shadow; D, airspace; H, hyoid bone and its acoustic shadow; T, tongue; HM, hyoid-external-meatus; RG, retro-glossal; RP, retro-palatal; UAL, upper airway length.

The US was done by an independent operator (Shu CC) who was blinded to the PSG results. The US machines used was Aplio XV (Toshiba Medical Systems, Tokyo, Japan) with 5.0-MHz convex transducers in gray scale 2-dimensional mode. The detailed procedure was shown in Figure 1.

Dynamic changes of transverse diameter of the retro-palatal (RP) and retro-glossal (RG) airspaces were determined in three maneuvers, namely, expiration during tidal breathing, forced inspiration, and the Müller maneuver (MM) (Fig. 1C). Before the measurement of dynamic changes, participant had time to practice the maneuvers under instruction. Forced inspiration was meant to approach the inspiratory reserve volume by maximal effort of inspiration within 3 sec, whereas MM was performed by an attempt at vigorous inhalation with the mouth and nose closed [21]. To localize measurement, two strategies were applied. First, no shoulder and head movement was allowed during the maneuver. Second, we measured the airspace 3 times in every participant based on the same level of US-structure composed of tongue, hypoglossal muscle and geniohyoid muscle (Fig. 1B&C). The percentages (%) of RP and RG shortening diameters were calculated by the percentage of pharyngeal diameter shortening from the diameter upon expiration during tidal breathing to that during forced inspiration or MM. Tongue thickness was measured at the coronal view (Fig. 1D), while the upper airway length (UAL) was measured from the anterior edge of the hyoid bone to the posterior edge of the hard palate, the sagittal plane (Fig. 1E.), which was modified from Segal's study [22].

The intra- and inter-observer coefficients of variation (CV) were tested on eight healthy volunteers where US was done by two independent operators (Shu CC and Huang CT). The intra- and inter-observer CVs ranged from 2.3 (tongue thickness) to 9.1 (forced inspiration of RP diameter) and 3.0 (tongue thickness) to 10.4 (expiration of RG diameter), respectively (Table S1). Moreover, US was repeated three times in 20 randomly recruited OSA patients and the intra-observer CV for RP diameter was 7.5 on expiration, 6.4 on forced inspiration, and 8.3 on MM.

### Statistical analysis

Inter-group differences for numerical variables were compared using student *t* test or one-way ANOVA, where appropriate, and the *chi*-square test for categorical variables. Factors correlated with AHI were identified by Pearson correlation. Multivariate logistic regression with forward conditional method was used to identify predictors for severe OSA. Thereafter, these predictors were used to estimate the patient's probability of severe OSA, which was then used to test the receiver operating characteristic (ROC) curve of diagnosing severe OSA. The optimal cut-off value of the ROC curve was defined as the one with the least (1 - sensitivity)^2^+ (1- specificity)^2^ in the model-development group [23]. This was then tested in the validation group. A two-sided *p*<0.05 was considered statistically significant. All statistical analyses were performed using the SPSS 13.0 (SPSS Inc., Chicago, IL, USA).

## Results

**Figure 2:**
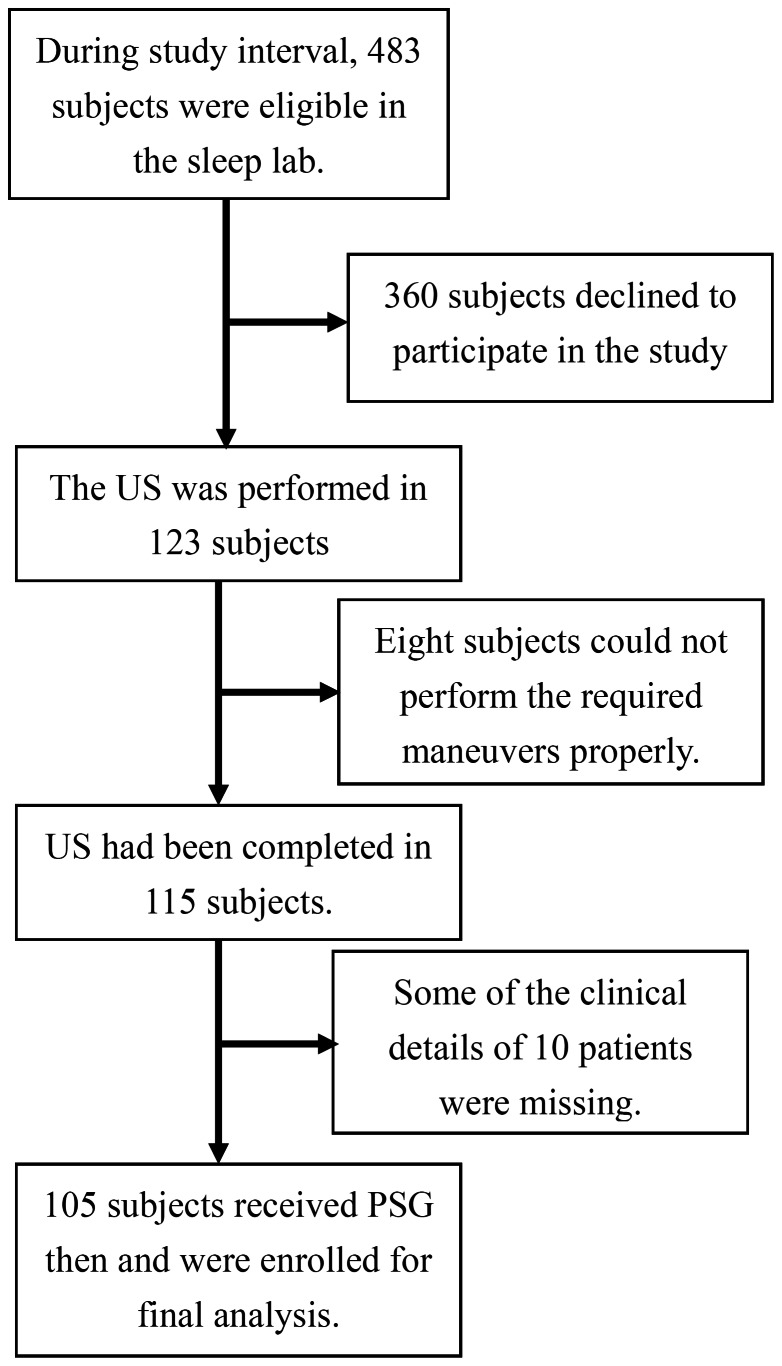
Flowchart of the trial.

The trial profile was listed in Figure 2. Among 483 eligible patients, 123 were enrolled and 115 completed the study. Among the 115 patients, clinical data was incomplete in 10, including missing body weight in three and neck circumference in seven. Data from the 105 patients were analyzed, with 25, 30, and 50 patients as non-OSA, mild-moderate, and severe OSA, respectively. Their demographics were shown in Table 1. Compared to non-OSA and mild-moderate OSA patients, severe OSA patents were older, more obese, and had larger neck circumference.

**Table 1:** Demographics of participants with no OSA, mil-moderate OSA, and severe OSA (n = 105).

	No OSA (n = 25)	Mild-moderate OSA (n = 30)	Severe OSA (n = 50)	*p*
Age (years)	38.2±12.1	41.4±12.6	49.2±14.4	0.002
Male, n (%)	20 (80%)	22 (73%)	45 (90%)	0.145
Body mass index (kg/m^2^)	22.7±2.8	25.7±5.5	28.2±4.0	<0.001
Neck circumference (cm)	35.9±2.9	37.4±4.2	41.1±3.7	<0.001
AHI (/hr)	2.4±1.5	16.5±7.5	59.4±16.3	<0.001

Abbreviation: AHI, Apnea-hypopnea index.

Data are mean ± SD unless otherwise indicated; *p* value was obtained by comparing three groups by one-way ANOVA.

The US parameters were shown in Table 2. The RP diameter was shortest in severe OSA and longest in the non-OSA on expiration during tidal breathing, forced inspiration, and MM. The RG diameter was narrowest in severe OSA during forced inspiration and MM. For dynamic changes, the % RP and % RG shortening on forced inspiration and MM were also much more in the severe OSA group than in the non-OSA and mild-moderate OSA groups. Three patients with mean AHI 75.4/hr had total collapse of the RP region during MM. Moreover, the severe OSA group had the thickest tongue and longest UAL compared to the non-OSA and mild-moderate OSA groups (Table 2).

**Table 2:** Ultrasonographic parameters of non-OSA, mild-moderate OSA, and severe OSA patients (n = 105).

	No OSA (n = 25)	Mild-moderate OSA (n = 30)	Severe OSA (n = 50)	*p*
Tongue thickness (mm)	40.3±5.9	43.1±5.0#	47.5±7.6	<0.001
Upper airway length (mm)	54.9±6.2	57.0±7.3#	61.8±5.9	<0.001
**Retro-palatal diameter**				
Expiration (mm)	36.6±7.7*	29.2±7.0	27.4±6.5	<0.001
Forced inspiration (mm)	33.2±7.5*	25.2±7.5	22.4±6.0	0.001
Müller maneuver (mm)	29.2±6.2*	19.8±6.6#	14.0±7.2	<0.001
Change at forced inspiration (%)	9.6±4.2*	14.6±9.8	18.4±8.7	0.012
Change at Müller maneuver (%)	20.9±8.1*	34.9±9.3#	50.0±19.6	<0.001
**Retro-glossal diameter**				
Expiration (mm)	38.9±4.3	37.3±5.2	36.7±6.2	0.251
Forced inspiration (mm)	35.1±4.6*	32.3±4.8	31.7±6.5	0.045
Müller maneuver (mm)	29.4±4.7*	25.3±5.0	22.7±8,3	<0.001
Change at forced inspiration (%)	9.9±4.5*	13.2±5.7	12.5±6.7	0.037
Change at Müller maneuver (%)	24.8±8.9	28.3±7.5	33.5±16.5	0.019

Data are mean ± SD; *p* value for inter-three-group comparison was obtained by one-way ANOVA.

* Significant *p* value (<0.05) between non-OSA and mild-moderate OSA groups using independent student *t* test.

# Significant *p* value (<0.05) between mild-moderate OSA and severe OSA groups using independent student *t* test.

Factors correlated with AHI included old age, male sex, body mass index (BMI), neck circumference, UAL, tongue thickness, RP diameter in three maneuvers, and RG diameter on forced inspiration and MM (Table S2). Among them, neck circumference had the highest Pearson correlative coefficient (γ = 0.659, *p*<0.001), followed by RP diameter on MM (γ = −0.624, *p*<0.001), UAL (γ = 0.581), % RP shortening on MM (γ = 0.584, *p*<0.001), and BMI (γ = 0.531, *p*<0.001).

By computer-generated randomization of the 105 patients with complete data, 70 were selected as the model-development group and 35 as the validation group. The model-development group included 15 non-OSA, 23 mild-moderate OSA, and 32 severe OSA patients. The validation group included 10 non-OSA, 7 mild-moderate OSA, and 18 severe OSA patients. The two groups had similar demographics and ultrasound parameters (Table 3). Multivariate analysis of the model-development group identified % RP shortening on MM (OR: 1.087, 95% CI: 1.022∼1.156; *p* = 0.008) and neck circumference (OR: 1.379, 95% CI: 1.138∼1.617; *p* = 0.001) as independent predictors of severe OSA (Table 4). With these two factors, the probability [Logit (P)] was generated by the formula: 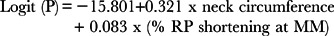
where the Logit (P) stood for log(P/1-P).

**Table 3:** Demographic and ultrasonographic parameters of the model-development and validation groups.

	Model-development group (n = 70)	Validation group (n = 35)	*p* *
Age, year	44.1±13.5	44.8±15.6	0.845
Male	57 (81%)	30 (86%)	0.583
BMI (kg/m^2^)	25.8±4.6	26.9±5.0	0.313
Neck circumference (cm)	38.5±4.5	39.3±3.8	0.305
AHI (episode/hour)	33.4±28.0	22.9±28.1	0.929
**Ultrasonographic parameters**			
Retro-palatal diameter			
Expiration (mm)	29.1±7.6	32.1±8.1	0.063
Forced inspiration (mm)	25.0±7.5	27.2±8.9	0.209
Müller maneuver (mm)	18.7±8.8	20.5±9.7	0.357
Change at forced inspiration (%)	14.7±8.2	16.3±10.1	0.406
Change at Müller maneuver (%)	39.2±18.4	37.4±20.0	0.670
Retro-glossal diameter	22.7±2.8	25.7±5.5	
Expiration (mm)	37.4±5.8	37.3±5.2	0.866
Forced inspiration (mm)	32.9±5.8	32.4±5.8	0.705
Müller maneuver (mm)	25.5±7.1	24.4±7.4	0. 474
Change at forced inspiration (%)	12.4±6.2	13.4±7.3	0.513
Change at Müller maneuver (%)	29.5±12.1	30.7±15.4	0.687
Tongue thickness (mm)	44.5±6.9	44.5±7.7	0.978
Upper airway length (mm)	58.3±7.3	59.8±6.3	0.302

Data are mean ± SD; *p* value for inter-group comparison was obtained by *t* test.

Abbreviations: AHI, apnea-hypopnea index; BMI, body mass index.

* Analyzed by independent student *t* test.

**Table 4:** Odds ratio for severe obstructive sleep apnea in the model-development group by univariate and multivariate analyses.

	Univariate			Multivariate		
	OR	95% C.I.	*p* value	OR	95% C.I.	*p* value
Age, year	1.038	1.000∼1.077	0.053			
Male	3.452	0.859∼13.87	0.081			
BMI (kg/m^2^)	1.354	1.147∼1.599	<0.001			0.191
Neck circumference (cm)	1.471	1.218∼1.776	<0.001	1.379	1.138∼1.671	0.001
Retro-palatal diameter						
Expiration (mm)	0.906	0.843∼0.974	0.007			0.666
Forced inspiration (mm)	0.882	0.816∼0.953	0.001			0.517
Müller maneuver (mm)	0.859	0.793∼0.931	<0.001			0.950
Change in forced inspiration (%)	1.099	1.027∼1.177	0.006			0.838
Change in Müller maneuver (%)	1.111	1.047∼1.178	<0.001	1.087	1.022∼1.156	0.008
Retro-glossal diameter						
Expiration (mm)	0.984	0.906∼1.068	0.701			
Forced inspiration (mm)	0.982	0.904∼1.066	0.665			
Müller maneuver (mm)	0.939	0.870∼1.012	0.101			
Change in forced inspiration (%)	1.017	0.942∼1.099	0.659			
Change in Müller maneuver (%)	1.032	0.988∼1.078	0.153			
Tongue Thickness (mm)	1.150	1.054∼1.256	0.002			0.682
Upper airway length (mm)	1.153	1.060∼1.253	0.001			0.587

Abbreviations: AHI, apnea-hypopnea index; BMI, body mass index; OR, odds ratio.

Significant variables (*p*<0.05) in univariate analysis were used for multivariate logistic regression analysis via forward conditional method.

The outline of probabilities was generated using the quartiles of neck circumference and % RP shortening on MM (Table 5). The optimal cut-off value for probability was 0.45 by the ROC curve.

**Table 5:** Probability of diagnosing severe obstructive sleep apnea in the model-development group*.

NC % RP shortening	31 cm	35 cm	38 cm	41 cm	52 cm
12%	<0.001	<0.001	0.002	0.022	0.987
28%	<0.001	0.005	0.049	0.326	1.000
35%	0.001	0.021	0.166	0.648	1.000
43%	0.005	0.091	0.480	0.894	1.000
100%	0.996	0.999	1.000	1.000	1.000

Abbreviations: NC, neck circumference; % RP during MM: percentage of changes in retro-palatal diameter change in Müller maneuver.

* Calculated by verifying the formula according to the quartiles of NC and % RP shortening during MM.

**Figure 3:**
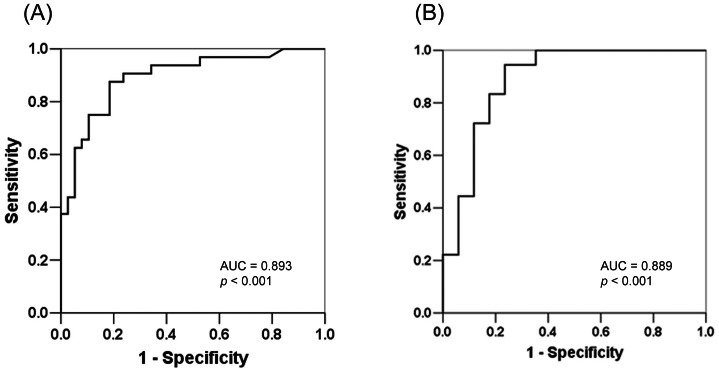
Receiver operating characteristic (ROC) curves for probability of severe OSA in the (A) model-development group and (B) validation group.

The ROC curve of the model-development group for identifying patients with severe OSA was shown in Figure 3. The area under the curve (AUC) was 0.893 (95% CI: 0.816–0.970, *p*<0.001) (Fig. 3A). When the probability was>0.45, severe OSA was predicted with 88% sensitivity and 92% specificity. The formula was also verified in the validation group. The AUC of ROC was 0.889 (95% CI: 0.774–1.004, *p*<0.001). When the probability was>0.45, severe OSA was predicted with 100% sensitivity and 65% specificity.

## Discussion

In this study, sub-mental US was used to measure the pharyngeal transverse diameter and nearby soft tissue in OSA patients. The pharyngeal variables, tongue thickness, and UAL had good correlation with OSA severity, especially RP diameter on MM. To predict severe OSA, % RP shortening during MM and neck circumference were used to construct a prediction model. The model was validated in an independent group where it had high sensitivity (100%) and fair specificity (65%).

The prevalence rate of OSA is as high as 24% in middle-aged men and 9% in middle-aged women [24]. A non-invasive, radiation-free, and relatively less expensive screening tool is needed to identify patients with severe OSA who require earlier intervention [6]. Among the tests, the OSA questionnaire and nocturnal pulse oximetry are two of the most common screening methods used. However, US is more objective than the questionnaire, more convenient because it does not require overnight time consumption, and more relevant than pulse oximetry for examining the pharyngeal airspace. This is supported by the very good sensitivity of the verifying formula in the present study, which demonstrates that sub-mental US is sufficiently sensitive for differentiating OSA severity with reference to RP diameters.

The US is also adequately sensitive for detecting dynamic changes of the pharyngeal airspace in three maneuvers, which means that like fiberoscopy and ultra-fast MRI, sub-mental US can demonstrate airway collapsibility and detect pharyngeal changes at apnea [15], [21], [22]. Although sub-mental US measures the low RP region instead of the velo-pharynx, the critical region of upper airway obstruction in OSA, its high sensitivity and specificity corroborates that the US-measured RP region is a good landmark for screening severe OSA patients. In addition, the US can only measure the transverse pharyngeal dimension but not the antero-posterior (AP) diameter but, compared to normal subjects, the transverse dimension is narrower in OSA patients while the AP dimension remains unchanged [21], [25].

The tongue base and lateral pharyngeal wall measured by US are shown to have good correlations with AHI in OSA patients [17], [19]. The present study explores tongue thickness and UAL aside from pharyngeal anatomy and the results show that UAL significantly correlated with AHI (γ = 0.499), which is similar to the level detected by CT (γ = 0.406) [22]. Thus, by integrating the information of laryngeal space and adjacent soft-tissue, sub-mental US can provide a complete picture of dynamic changes of the upper airway.

It has been demonstrated that the RP region is the primary site of upper airway narrowing in OSA patients in both Caucasians and Asians when compared to the RG region [26]. Compared to Caucasians, Chinese patients have similar upper airways but larger supero-posterior airways, nasopharynx, and oropharynx [27]. Therefore, the high sensitivity of sub-mental US in identifying patients with severe OSA may also be applicable for Caucasian patients.

The present study has some limitations. First, a pharyngeal pressure monitor was not used to monitor the effects of the Müller maneuver. Although all enrolled patients cooperated and performed the maneuver well, its effect of MM might not be the same for all. Second, there was no MRI used to compare the anatomic markers detected by US.

In conclusion, sub-mental US can accurately measure pharyngeal airspace and adjacent soft tissue, and can discriminate OSA severity. The % RP shortening during MM and neck circumference are independent factors for predicting severe OSA. Thus, sub-mental US may be a promising tool for screening severe OSA patients for early intervention.

## Supporting Information

File S1 **Recruited population for examining intra- and inter-observer variation**.
(DOC)

Table S1 **Coefficients of variance of pharynx measured by ultrasonography.**
(DOC)

Table S2 **Correlations between clinical and ultrasonographic variables and apnea-hypopnea index in the study patients.**
(DOC)
