# Supplementary material for: The Use of Sub-Mental Ultrasonography for Identifying Patients with Severe Obstructive Sleep Apnea
Source: PLoS One. 2013 May 10;8(5):e62848. doi: 10.1371/journal.pone.0062848 (PMC3651088; doi:10.1371/journal.pone.0062848)
Supplement: File S1 — Recruited population for examining intra- and inter-observer variation. (DOC) [file pone.0062848.s001.doc]

**Recruited population for examining intra- and inter-observer variation**

The 8 volunteers without OSA symptoms were recruited for examination with regards to intra- and inter-observer variation. They were on average 33.5 years-old and their body mass index (BMI) and male gender ratio were 24.0 and 0.63, respectively. Then, we randomly selected 20 subjects with OSA symptoms that were enrolled in our study to to undergo an intra-observer variation check.

The 20 subjects had a mean age of 50.3 years, mean body mass index of 27.7 and a male gender ratio of 0.7. Ten (50%) of them were categorized as belonging to the severe OSA group and the remaining were part of the mild-to-moderate OSA group. The data of intra- and inter-observer variation are summarized in Table S1.
