# Supplementary material for: The Use of Sub-Mental Ultrasonography for Identifying Patients with Severe Obstructive Sleep Apnea
Source: PLoS One. 2013 May 10;8(5):e62848. doi: 10.1371/journal.pone.0062848 (PMC3651088; doi:10.1371/journal.pone.0062848)
Supplement: Table S1 — Coefficients of variance of pharynx measured by ultrasonography. (DOC) [file pone.0062848.s002.doc]

**Table S1.** Coefficients of variance (CV) of pharynx measured by ultrasonography (n=8).

|  | Intra-observer CV  (Volunteers, n=8) | Inter-observer CV  (Volunteers, n=8) | Intra-observer CV  (Patients, n=20) |
| --- | --- | --- | --- |
| Retro-palatal diameter |  |  |  |
| Expiration | 4.5 ± 3.3 | 3.4 ± 2.6 | 7.5 ± 3.3 |
| Forced inspiration | 9.1 ± 5.6 | 6.8 ± 3.2 | 6.4 ± 4.3 |
| Müller maneruver | 8.6 ± 5.6 | 9.0 ± 6.4 | 8.3 ± 5.4 |
| Retro-glossal diameter |  |  |  |
| Expiration | 3.6 ± 2.2 | 10.4 ± 3.6 | 5.9 ± 4.1 |
| Forced inspiration | 6.3 ± 5.7 | 7.3 ± 6.2 | 6.9 ± 3.3 |
| Müller maneruver | 7.9 ± 5.5 | 8.8 ± 7.7 | 9.3 ± 6.3 |
| Tongue thickness | 2.3 ± 1.3 | 3.0 ± 3.1 |  |
| Upper aiway length | 3.0 ± 1.6 | 4.3 ± 2.0 |  |

All data are shown as mean ± standard deviation
