# Supplementary material for: The Use of Sub-Mental Ultrasonography for Identifying Patients with Severe Obstructive Sleep Apnea
Source: PLoS One. 2013 May 10;8(5):e62848. doi: 10.1371/journal.pone.0062848 (PMC3651088; doi:10.1371/journal.pone.0062848)
Supplement: Table S2 — Correlations between clinical and ultrasonographic variables and apnea-hypopnea index in the study patients. (DOC) [file pone.0062848.s003.doc]

**Table S2. Correlations between clinical and ultrasonographic variables and apnea-hypopnea index in the study patients (n=105)**

|  | Correlation with AHI | *p* value* |
| --- | --- | --- |
| Age, year | 0.276 | 0.004 |
| Male gender | 0.205 | 0.036 |
| BMI (kg/m^2^) | 0.531 | < 0.001 |
| Neck circumference (cm) | 0.659 | < 0.001 |
| **Ultrasonographic parameters** |  |  |
| Retro-palatal diameter |  |  |
| Expiration (mm) | - 0.435 | < 0.001 |
| Forced inspiration (mm) | - 0.515 | < 0.001 |
| Müller maneuver (mm) | - 0.624 | < 0.001 |
| Change in forced inspiration (%) | 0.455 | < 0.001 |
| Change in Müller maneuver (%) | 0.584 | < 0.001 |
| Retro-glossal diameter |  |  |
| Expiration (mm) | - 0.142 | 0.149 |
| Forced inspiration (mm) | - 0.225 | 0.021 |
| Müller maneuver (mm) | - 0.348 | 0.001 |
| Change in forced inspiration (%) | 0.278 | 0.004 |
| Change in Müller maneuver (%) | 0.254 | 0.009 |
| Tongue thickness (mm) | 0.474 | < 0.001 |
| Upper airway length (mm) | 0.499 | < 0.001 |

Abbreviations: AHI, apnea-hypopnea index; BMI, body mass index

*Correlation analyzed by Pearson’s coefficient method
